# Generation of Trophoblast-Like Cells From Hypomethylated Porcine Adult Dermal Fibroblasts

**DOI:** 10.3389/fvets.2021.706106

**Published:** 2021-07-19

**Authors:** Sharon Arcuri, Georgia Pennarossa, Fulvio Gandolfi, Tiziana A. L. Brevini

**Affiliations:** ^1^Laboratory of Biomedical Embryology, Department of Health, Animal Science and Food Safety and Centre for Stem Cell Research, UniStem, Università Degli Studi di Milano, Milan, Italy; ^2^Laboratory of Biomedical Embryology, Department of Agricultural and Environmental Sciences-Production, Landscape, Agroenergy and Centre for Stem Cell Research, UniStem, Università Degli Studi di Milano, Milan, Italy

**Keywords:** 5-azacytidine, epigenetic conversion, fibroblasts, porcine, trophoblast-like cells

## Abstract

The first differentiation event in mammalian embryos is the formation of the trophectoderm, which is the progenitor of the outer epithelial components of the placenta, and which supports the fetus during the intrauterine life. However, the epigenetic and paracrine controls at work in trophectoderm differentiation are still to be fully elucidated and the creation of dedicated *in vitro* models is desirable to increase our understanding. Here we propose a novel approach based on the epigenetic conversion of adult dermal fibroblasts into trophoblast-like cells. The method combines the use of epigenetic erasing with an *ad hoc* differentiation protocol. Dermal fibroblasts are erased with 5-azacytidine (5-aza-CR) that confers cells a transient high plasticity state. They are then readdressed toward the trophoblast (TR) phenotype, using MEF conditioned medium, supplemented with bone morphogenetic protein 4 (BMP4) and inhibitors of the Activin/Nodal and FGF2 signaling pathways in low O_2_ conditions. The method here described allows the generation of TR-like cells from easily accessible material, such as dermal fibroblasts, that are very simply propagated *in vitro*. Furthermore, the strategy proposed is free of genetic modifications that make cells prone to instability and transformation. The TR model obtained may also find useful application in order to better characterize embryo implantation mechanisms and developmental disorders based on TR defects.

## Introduction

Reproduction in eutherian mammals strictly depends on the placenta that is required for both maintenance of pregnancy and fetal development. The placenta is a transient extraembryonic organ that nourishes and supports the fetus during intrauterine life. It anchors the fetus to the uterine wall, provides immune protection and ensures transport and exchange of nutrients, gases, waste and hormones between the mother and the fetus (1). Trophoblast (TR) cells represent the major placental cell type and are highly specialized in each of the above mentioned functions (2, 3). These cells originate from a simple epithelial sheet that surrounds the blastocoel and the inner cell mass (ICM) at the blastocyst stage, namely the embryonic trophectoderm (TE). Comparative studies on placental morphology revealed that TR tissue can range from a single layer of cells to a complex multilayered organization, based on the different types of placentation (4). Specifically, in species exhibiting hemochorial placentation, such as the murine and the human, during embryo implantation, the outer trophectoderm layer attaches to the uterine endometrial epithelial cells and differentiates into the two main subpopulations, namely cytotrophoblast (CT) and syncytiotrophoblast (ST) cells. The former directly derives from trophoblast stem cells and constitutes an undifferentiated non-polarized proliferative cell population. CT cells are considered the major invasive component of the placenta, since they aggregate into multilayered columns at the tips of villi, forming their basement membrane, and migrate from this site into the maternal endometrium, through both the uterine stroma and the lumen of maternal arteries (5). In addition, in hemochorial placentae, villous CT cells fuse together and differentiate into multinucleated ST cells (6, 7), which cover the villi and make direct contact with maternal blood, becoming the major site of maternal–fetal gas and nutrient exchange (8). Conversely, animals with non-invasive epitheliochorial placentation, namely horses, camels, pigs, hippopotami, and cetaceans, do not form an ST layer (9) and CT cells directly interact with maternal uterine luminal epithelium, forming the trophoblast-endometrial epithelial bilayer (also known as “interareolar region”) and serving analogous functions of nutrition and gas exchange (10, 11).

To date, TR cells have been extensively described in the mouse (12, 13), where they can be easily derived from blastocysts or from extra-embryonic ectoderm (14). In contrast, a more limited amount of information is available in large mammals where they can be isolated with poor efficiency (15, 16). Furthermore, although the transcriptional regulation driving TR cell differentiation processes are beginning to be understood, experimental limitations exist to investigate the early events taking place during the first stages of pregnancy (2, 17). The creation of a large mammal *in vitro* model that mimics the initial steps of placenta development is therefore needed in order to increase our understanding and better elucidate both the epigenetic mechanisms and the paracrine controls at work during TR specification (18). It is interesting to note that porcine induced TR cells spontaneously generated during standard reprogramming processes (19) and formed floating spheres consisting of a single epithelial sheet, whose cells expressed transcription factors associated with TR lineage (20).

Here we describe an alternative approach based on the epigenetic conversion of adult porcine dermal fibroblasts into TR-like cells. The method combines the use of the epigenetic eraser 5-azacytidine (5-aza-CR) that pushes fibroblasts toward a less committed high plasticity state. Erased cells are then readdressed toward the TR lineage, using MEF conditioned medium supplemented with bone morphogenetic protein 4 (BMP4) and inhibitors of the Activin/Nodal and FGF2 signaling pathways.

## Materials and Methods

All reagents were purchased from Thermo Fisher Scientific unless otherwise indicated.

### Ethical Statement

This study was authorized by the Ethical Committee of the University of Milan, Italy. All methods were carried out in accordance with the approved guidelines.

### Isolation and Culture of Adult Porcine Dermal Fibroblasts

Porcine dermal fibroblasts were isolated from fresh abdominal skin biopsies of 3 female and 2 male adult individuals. Tissues were cut in small fragments of ~2 mm^3^, transferred onto 0.1% gelatin (Sigma) pre-coated Petri dishes (Sarstedt) and cultured in Dulbecco's modified Eagle's medium (DMEM) supplemented with 20% Fetal Bovine Serum (FBS), 2 mM glutamine (Sigma) and antibiotics. After 6 days of culture, fibroblasts started to grow out of the explant fragments and the latter were carefully removed. One primary cell line was established from each skin biopsy. Fibroblasts were maintained in the medium described above, grown in 5% CO_2_ at 37°C and passaged twice a week in a 1:3 ratio. Primary cell lines obtained from each individual (*n* = 5) were used at least in triplicate in 3 independent experiments.

### Treatment of Adult Porcine Dermal Fibroblasts With 5-Aza-CR

Fibroblasts at passages between 6 and 8 after isolation were plated in 4-well multidishes (Nunc) previously coated with 0.1% gelatin (Sigma) at a concentration of 7.8 × 10^4^ cells/cm^2^. Twenty-four hours after seeding, cells were exposed to the epigenetic eraser 5-aza-CR (Sigma) at 1 μM concentration for 18 h. Concentration and time of exposure were selected based on our previous studies (21–27).

### Trophoblast Induction

Post 5-aza-CR treatment, cells were cultured in embryonic stem cell (ESC) medium consisting of DMEM-low glucose:HAM'S F10 (1:1) supplemented with 5% FBS, 10% K.O. serum, 2 mM glutamine, 0.1 mM β-mercaptoethanol, nucleoside mix, 1% non-essential amino acids, 1,000 IU/ml ES-growth (LIF, Chemicon), 5 ng/ml b-FGF (R&D System) (22, 28, 29) for 3 h at 37°C in 5% CO_2_. Culture dish were then incubated at 37°C in low O_2_ condition (5% O_2_, 5% CO_2_, and 90% N_2_ atmosphere) for 21 h. TR differentiation was induced using mouse embryonic fibroblast (MEF) conditioned medium obtained by culturing inactivated MEF in ESC culture medium without b-FGF for 24 h (see below) supplemented with 10 ng/ml bone morphogenetic protein 4 (BMP4, Sigma), 1 μM activin/nodal signaling inhibitor (A83-01, Sigma) and 0.1 μM FGF2 signaling inhibitor (PD173074, Sigma) (30). Cells were maintained in low O_2_ conditions at 37°C and culture medium was refreshed every other day, until day 11, when culture was arrested. Phenotype induction was daily scored using a Nikon Eclipse TE200 microscope and specific analysis were carried out at day 2, 5, and 11, in agreement with previously published results (2, 30).

### Preparation of MEF Conditioned Medium

MEFs were routinely cultured in complete MEF medium consisting of DMEM supplemented with 10% FBS, 2 mM glutamine and antibiotics. Once confluence was reached, cells were plated at a seeding density of 20.000 cells/cm^2^. Twenty-four hours after plating, cells were inactivated with 10 μg/ml mitomycin-C for 3 h and subsequently cultured in ESC culture medium without b-FGF for 24 h. Conditioned medium was harvested, filtered, and stored at −20°C till further use.

### Global DNA Methylation Analysis

Genomic DNA was extracted using the PureLink^®^ Genomic DNA Kit following the manufacturer's protocol. Double-stranded DNA was denatured by incubation at 95°C for 5 min, followed by rapid chilling on ice. It was digested to nucleosides using nuclease P1 in 20 mM sodium acetate (pH 5.2) for 2 h at 37°C. Alkaline phosphatase in 100 mM Tris (pH 7.5) was added and an 1 h incubation at 37°C was performed. Samples were centrifuged and the supernatant was used for ELISA assay using Global DNA Methylation ELISA Kit (5′-methyl-2′-deoxycytidine Quantitation; CELL BIOLABS) according to the manufacturer's instructions.

### Gene Expression Analysis

RNA was extracted using the TaqManGene Expression Cells to Ct kit (Applied Biosystems) and DNase I was added in lysis solution at 1:100 concentration, as indicated by the manufacturer's instructions. Quantitative PCR was performed using the CFX96 Real-Time PCR (Bio-Rad Laboratories) and pre-designed gene-specific primers and probe sets from TaqManGene Expression Assays (Table 1). *GAPDH* and *ACTB* were used as internal reference genes. Target gene quantification was carried out with CFX Manager software (Bio-Rad Laboratories).

**Table 1:** List of primers used for quantitative PCR analysis.

**Gene**	**Description**	**Catalog No**.
ACTB	Actin beta	Ss03376563_uH
CDX2	Caudal type homeobox 2	Ss03373636_m1
CYP11A1	Cytochrome P450 family 11 subfamily A member 1	Ss03384849_u1
GAPDH	Glyceraldehyde-3-phosphate dehydrogenase	Ss03375435_u1
GCM1	Glial cells missing transcription factor 1	Ss03373780_m1
HSD17B1	Hydroxysteroid 17-beta dehydrogenase 1	Ss04245960_g1
IFNG	Interferon gamma	Ss03391052_m1
NANOG	Nanog homeobox	Ss04245375_s1
OCT4	POU class 5 homeobox 1	Ss03389800_m1
PAG6	Pregnancy-associated glycoprotein 6	Ss03378057_u1
PPAG3	Pregnancy-associated glycoprotein 3	Ss03392369_m1
REX1	ZFP42 zinc finger protein	Ss03373622_g1
SOX2	Sex determining region Y-box 2	Ss03388002_u1
THY1	Thy-1 cell surface antigen	Ss03376963_u1
VIM	Vimentin	Ss04330801_gH

### Immunocytochemical Analysis

Cells were fixed in 4% paraformaldehyde in PBS for 20 min at room temperature, washed three times in PBS and permeabilized with 0.1% Triton X-100 (Sigma) in PBS for 30 min. Samples were then treated with a blocking solution containing 10% goat serum (Sigma) in PBS for 30 min. Primary antibodies were incubated overnight at +4°C (for working dilutions see Table 2) followed by staining with suitable secondary antibodies (1:250, Alexa Fluor) for 1 h at room temperature. Nuclei were counterstained with 4′,6-diamidino-2- phenylindole (DAPI, Sigma). Samples were observed under a Nikon Eclipse TE200 microscope.

**Table 2:** List of antibodies and working dilutions used for immunocytochemical analysis.

**Antibody**	**Description**	**Company**	**Cat. No**.	**Working dilution**
CDX2	Rabbit monoclonal	Cell signaling	12306	1:50
KRT7	Mouse monoclonal	Santa cruz	sc-23876	1:100
HSD17B1	Rabbit polyclonal	Sigma-aldrich	AV41727	1:100
INFG	Rabbit monoclonal	Cell signaling	8455	1:100
NANOG	Rabbit polyclonal	Cell signaling	3580	1:500
OCT4	Rabbit polyclonal	Abcam	ab137427	1:200
VIM	Mouse monoclonal	Abcam	ab8978	1:100

### Cell Counting

The number of CDX2, KRT7, HSD17B1, and INFG immuno-positive cells was counted in 10 randomly selected fields at 200X total magnification. A minimum of 500 cells were scored in three independent replicates. The number of positively stained cells was expressed as a percentage of the total cell counted.

### Statistical Analysis

Statistical analysis was performed using the Student *t*-test (SPSS 19.1; IBM). Data were presented as mean ± standard deviation (SD). Differences of *p* ≤ 0.05 were considered significant and were indicated with different superscripts. The data were presented as mean of 3 separate experiments performed using triplicates of each of the 5 primary cultures isolated.

## Results

### Characterization of Adult Porcine Dermal Fibroblasts

**Figure 1:**
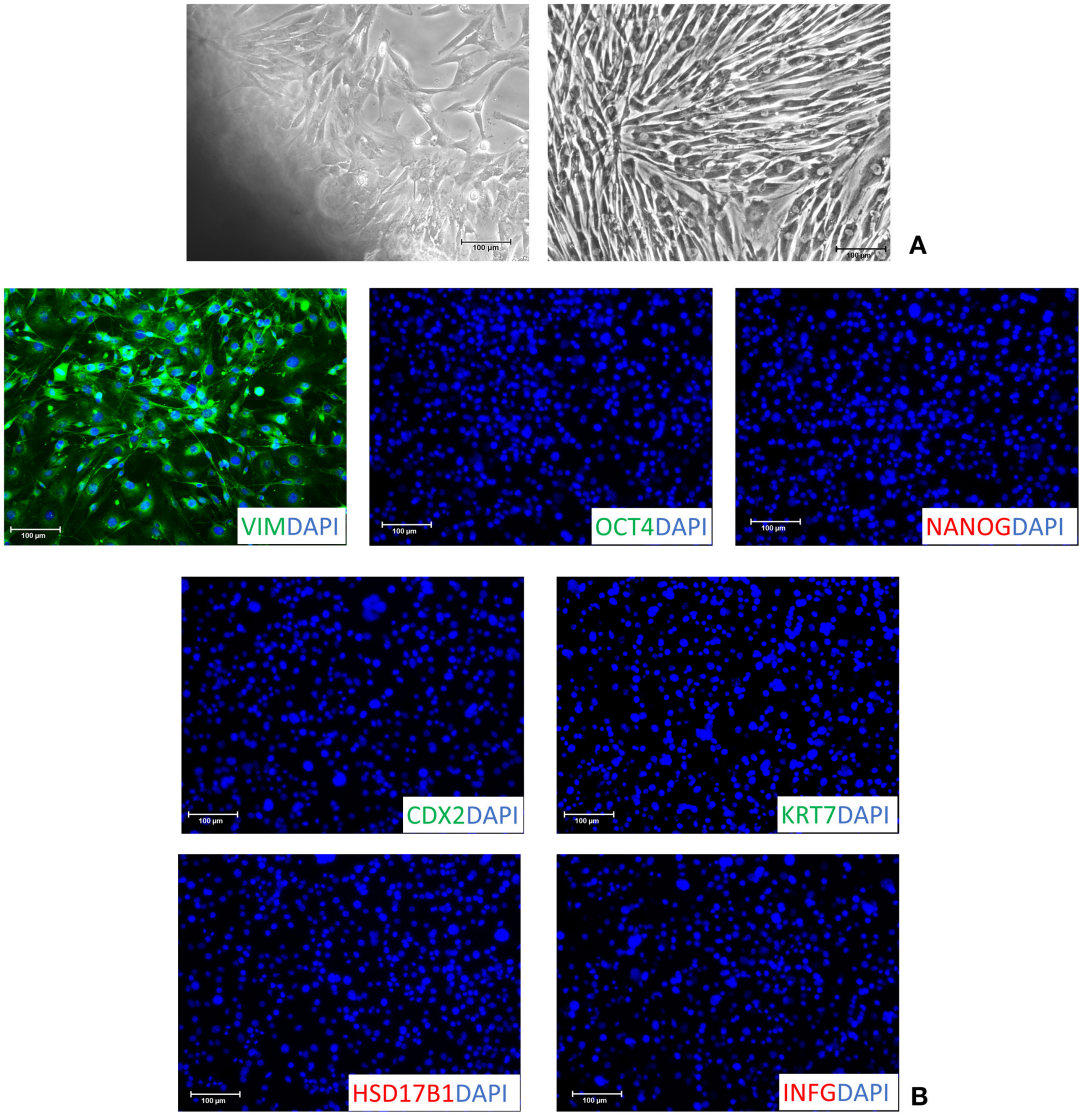
Characterization of adult porcine dermal fibroblasts. **(A)** Fibroblasts grew out of dermal tissue fragments within 6 days (left panel) and formed a monolayer (right panel). Cells displayed a standard morphology elongated in shape (right panel). Scale bars: 100 μm. **(B)** Isolated fibroblasts showed a uniform immuno-positivity for vimentin (VIM) and the complete absence of the pluripotent- (OCT4 and NANOG) and TR- (CDX2, KRT7, HSD17B1 and INFG) related markers. Nuclei were stained with DAPI (Scale bars: 100 μm).

**Figure 2:**
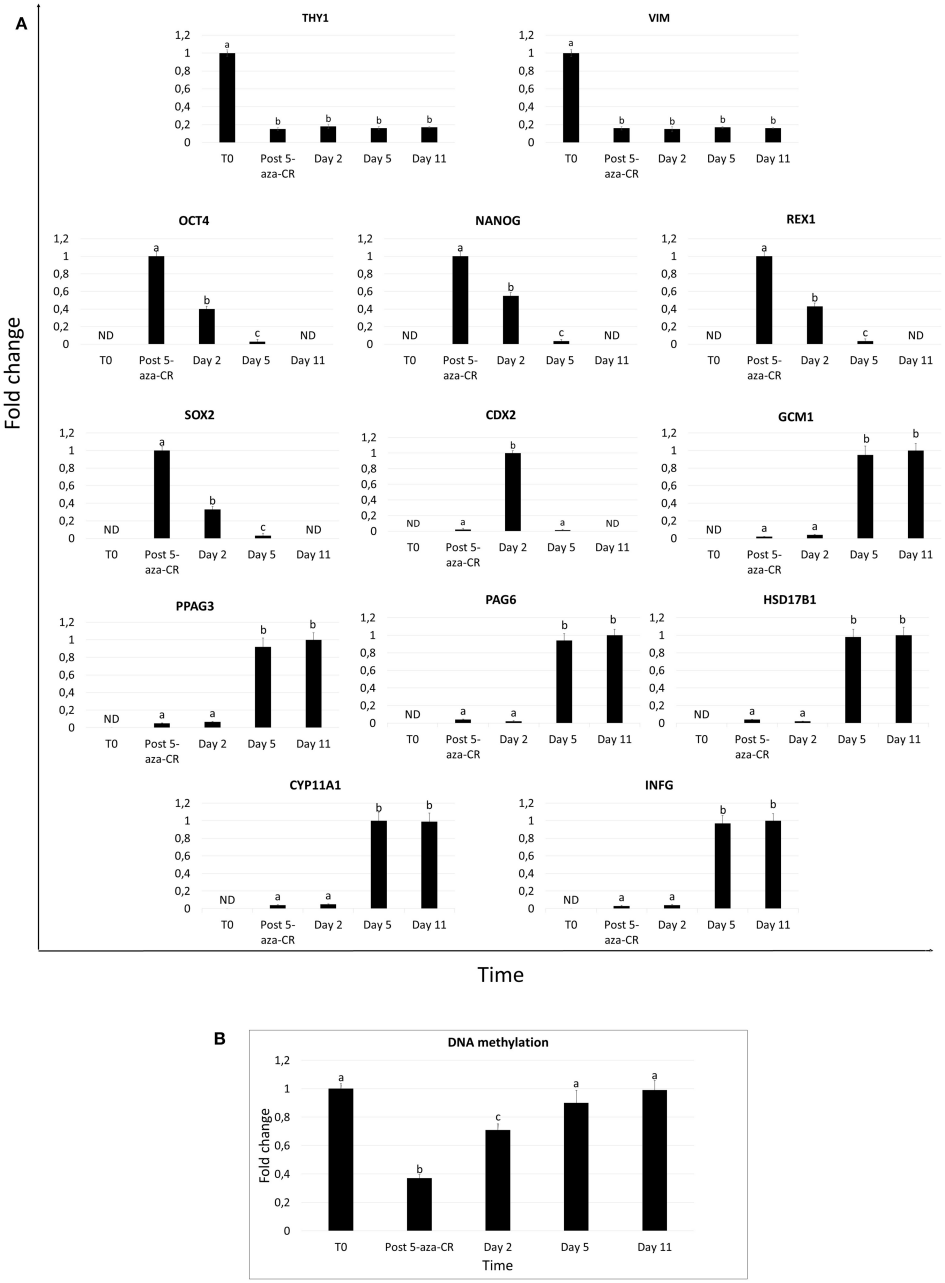
Gene expression profile and DNA methylation changes in adult porcine dermal fibroblasts exposed to 5-aza-CR and subjected to TR induction. **(A)** Expression pattern of fibroblast- specific (THY1, VIM), pluripotency-related (OCT4, NANOG, REX2, and SOX2), and early (CDX2) and mature TR (GCM1, PPAG3, PAG6, HSD17B1, CYP11A1, and IFNG) markers in untreated fibroblasts (T0), in fibroblast exposed to 5-aza-CR (Post 5-aza-CR), and at different days of TR induction (day 2, 5, and 11). Values are reported with highest expression set to 1 and all other times relative to this. Different superscripts denote significant differences (*P* ≤ 0.05). **(B)** Global DNA methylation levels in untreated fibroblasts (T0), in cells exposed to 5-aza-CR (Post 5-aza-CR) and during TR induction period (day 2, 5, and 11). Highest level set to 1 and all other relative to this. Bars represent the mean ± SD of three independent replicates. Different superscripts (a,b,c) denote significant differences (*P* ≤ 0.05).

Fibroblasts obtained from skin biopsies grew out of the original explants within 6 days of culture (Figure 1A, left panel) and formed a monolayer (Figure 1A, right panel). Cells displayed the standard morphology, elongated in shape, and a uniform immunopositivity for the fibroblast specific marker vimentin (VIM, Figure 1B). No signal for the pluripotency-related (OCT4 and NANOG) and the early (CDX2) and mature TR (KRT7, HSD17B1 and INFG) markers was detected (Figure 1B). In agreement with this, molecular analysis of untreated fibroblasts (T0) demonstrated a strong expression of the THY and VIM genes (Figure 2A), while pluripotency-related transcripts (OCT4, NANOG, REX1, and SOX2) were undetectable (Figure 2A). No expression was found for TR specific genes, namely CDX2, GCM1, PPAG3, PAG6, HSD17B1, CYP11A1, and IFNG (Figure 2A).

### Effect of 5-Aza-CR Exposure on Cell Plasticity

**Figure 3:**
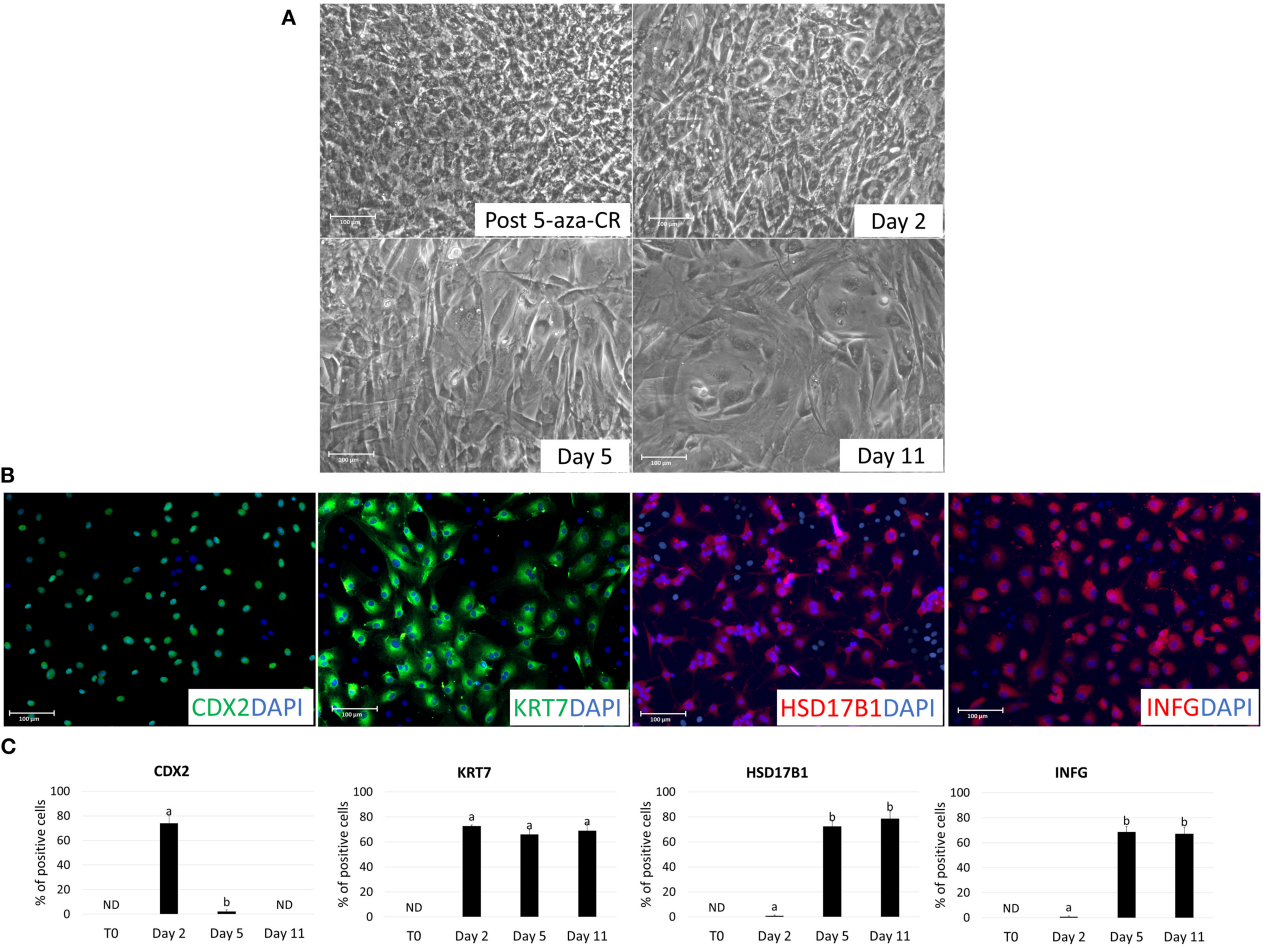
Morphological changes in adult porcine dermal fibroblasts after 5-aza-CR exposure and during TR induction. **(A)** Fibroblasts lost their typical elongated shape and become smaller with larger nuclei and granular cytoplasm in response to 18 h exposure to 5-aza-CR (Post 5-aza-CR). At day 2 of TR induction, cells acquired a tight adherent epithelial morphology (Day 2). By day 5 of differentiation, TR-like cells showed mature phenotype exhibiting round or ellipsoid shape with round nuclei and well-defined borders (Day 5 and 11). Scale bars: 100 μm **(B)** TR-like cells displayed immuno-positivity for early (CDX2) and mature TR (KRT7, HSD17B1, and IFNG) markers. Nuclei were stained with DAPI. Scale bars: 100 μm. **(C)** CDX2, KRT7, HSD17B1, and IFNG immunopositive cell rates in untreated fibroblasts (T0) and at different days of TR induction (day 2, 5, and 11). Bars represent the mean ± SD of three independent replicates. Different superscripts (a,b) denote significant differences (*P* ≤ 0.05).

After 5-aza-CR exposure, cell phenotype changed, and fibroblast elongated morphology was replaced by an oval or round shape. Treated cells became smaller with larger nuclei and granular and vacuolated cytoplasm (Post 5-aza-CR; Figure 3A). These changes were functionally accompanied by a significant decrease in global DNA methylation levels compared to untreated fibroblasts (T0, Figure 2B). In parallel, exposure to 5-aza-CR (Post 5-aza-CR) resulted in the onset of the pluripotency-related genes OCT4, NANOG, REX1, and SOX2, originally undetectable in untreated fibroblasts (T0; Figures 1B, 2A). In agreement with these observations, erased cells significantly downregulated the typical fibroblast markers THY and VIM (Figure 2A).

### Trophoblast Induction

After 2 days of culture with TR induction medium, cells acquired the tight adherent epithelial morphology typical of TR stem cells (Figure 3A). This change was accompanied by downregulation of the pluripotency-related genes (OCT4, NANOG, REX1, and SOX2; Figure 2A) and by an increase in global DNA methylation levels (Figure 2B). Consistent with this, active transcription of the early TR gene CDX2 was detected (Figure 2A). Immunocytochemical analysis confirmed this result, showing 74.05 ± 6.42% CDX2 and 72.8 ± 0.65% KRT7 immuno-positive cells (Figures 3B,C).

By day 5 of TR differentiation, cells acquired a mature TR morphology, with cells exhibiting round or ellipsoid shape, round nuclei, and well-defined borders (Figure 3A). These changes were paralleled by a further increase in DNA methylation levels, that returned comparable to those detected in untreated fibroblasts (T0) (Figure 2B). Onset of mature TR related genes, namely GCM1, PPAG3, PAG6, HSD17B1, CYP11A1, and IFNG, was observed (Figure 2A). The transcription of these markers was detectable at day 5 and stably maintained until day 11, when culture was arrested (Figure 2A). This was further support by immunocytochemical results, showing cell positivity for the mature TR markers KRT7, HSD17B1 and INFG, both at day 5 (65.99 ± 3.98%, 72.56 ± 4.1%, and 68.71 ± 4.25%, respectively) and at day 11 (69.1 ± 4.22%, 78.61 ± 5.56%, and 67.14 ± 5.47%, respectively) (Figures 3B,C). Interestingly, expression of the early TR marker CDX2 (Figure 2A) and detection of the encoded protein (Figure 3C) was downregulated by day 5.

## Discussion

In the present study we demonstrate the possibility to directly convert porcine adult dermal fibroblasts into TR-like cells, using the epigenetic eraser 5-aza-CR and an *ad hoc* lineage specific induction protocol. The possibility to generate *in vitro* cell colonies that display distinctive features of the TR lineage in the pig has been demonstrated before by Ezashi et al. (20). Here we further extend the feasibility to obtain a TR model in large mammalian species, starting from terminally differentiated cells.

5-aza-CR ability to reactivate silent genes and, as a consequence, modify the differentiation state of an eukaryotic cell, has been previously reported (31–33). Based on this, the epigenetic eraser has been extensively used to induce a high plasticity state in adult somatic cells by several authors (21–27, 34–41). In particular, the compound has been demonstrated to induce a global DNA hypomethylation, by actively modulating ten-eleven translocation (TET) gene transcription (27) and by indirectly inhibiting DNA methyltransferase (DNMT) activities (42). Consistent with these observations, in the present work, porcine adult dermal fibroblasts exposed to 5-aza-CR for 18 h, showed a significant decrease in global DNA methylation levels, compared to untreated fibroblasts (T0), suggesting the acquisition of a less committed state. Cell hypomethylation was paralleled by the onset of the main pluripotency-related gene transcription. In particular, OCT4, NANOG, REX1, and SOX2, which were originally undetectable in untreated fibroblasts (T0), were actively expressed after exposure to 5-aza-CR. Interestingly, the molecular changes described were supported by the morphological observations that showed the transition of the erased cell from the fibroblast typical elongated morphology to a round or oval shape, with larger nuclei and granular, vacuolated cytoplasm. These features closely resemble those previously identified in embryonic stem cells (ESCs) (43, 44) and induced pluripotent cells (iPS) (45), indicating the acquisition of morphological characteristics distinctive of a high plasticity phenotype.

Taking advantage of the acquired high permissivity window, cells were readdressed toward the TR lineage, using a well-established induction protocol that has been previously shown to successfully allow for the differentiation of human ESCs toward TR cells (30, 46–54). In particular, epigenetically erased fibroblasts were induced with MEF conditioned medium supplemented with BMP4 and inhibitors of the activin/nodal and FGF2 signaling pathways. The BMP is known to be involved in several differentiation processes, favoring the acquisition of multiple phenotypes belonging to both embryonic (55–57) and extraembryonic lineages (46, 58, 59). In addition, activin/nodal and FGF signaling have the ability to influence a broad range of BMP-mediated cellular events, including self-renewal and differentiation (46). In particular, the presence of FGF2, ACTIVIN-A, and BMP4 is able to drive lineage directionality in ESCs, selectively toward TR, mesoderm, or endoderm, depending on the relative concentration of each molecule in the culture medium (52). When FGF2 and activin-a/nodal signaling suppressors are added to the differentiation cocktail containing BMP4, the induction is reported to be completely and unidirectionally toward TR (2, 30, 52, 60). Although these observations were derived from experiments carried out with human cells, they are fully confirmed in the present work, where the use of appropriate concentrations of FGF2, ACTIVIN-A, and BMP4 allows for the generation of TR-like cells in the porcine specie. It must be however noted that the pig cell conversion efficiency obtained in our experiment resulted to be ~70%, which is lower than that scored in the human (higher than 90%) (30, 52, 61, 62). We have no clear explanation for this discrepancy. We hypothesize that species-specific differences may account for dissimilar lineage amenability. In addition, in the present work adult differentiated cells were used as a starting material, while the results described for the human were obtained from ESCs.

Interestingly, a further key aspect of the protocol adopted was represented by the use of low O_2_ conditions that mimic the physiological hypoxic environment of uterine milieu, favoring the correct lineage specification. Previous studies demonstrated that O_2_ tensions profoundly affect how quickly cells differentiate in response to BMP4 (2, 63) and tune the expression of several key TR-related genes (30, 60). The results presented in the present manuscript are consistent with these observations and demonstrate that the combination of the appropriate medium formulation and O_2_ tension was indeed able to induce the acquisition of a TR-like phenotype. In particular, after 2 days of induction, cells acquired a tight adherent epithelial morphology, comparable to that described for murine and human TR stem cells (64, 65). These changes were paralleled by the onset of the early TR gene CDX2 transcription and by downregulation of the pluripotency-related genes (OCT4, NANOG, REX1, and SOX2). Notably, these results are in agreement with the transcription pattern described during *in vitro* differentiation of ESCs toward the TR lineage that also report CDX2 gene expression peaking at day 2 of induction (30, 66–68). Consistent with this, by day 5 of induction, epigenetically erased cells showed a downregulated CDX2 expression and active transcription for the TR mature markers KRT7, GCM1, PPAG3, PAG6, HSD17B1, CYP11A1, and IFNG, that were stably maintained until day 11, when culture was arrested. Progression toward the new TR phenotype was accompanied by an increase in DNA methylation levels, that returned comparable to those of untreated fibroblasts (T0), suggesting cell transition from an hypomethylated state toward a higher methylation status, distinctive of a differentiated cell population (69, 70). The acquisition of a mature TR-like phenotype was further confirmed by the morphological observations, demonstrating that, by day 5 of induction, cells exhibited a round epithelial shape, round nuclei and well-defined borders. These features closely resemble those reported in Assheton *in vivo* study on porcine placental tissues (71). In addition, the cell phenotype obtained at the end of di induction period were highly reminiscent of TR cells isolated from *in vitro*-produced porcine blastocysts and parthenotes (16).

## Conclusions

Altogether, our findings demonstrate that it is possible to convert terminally differentiated adult dermal fibroblasts into porcine TR-like cells. The use of a well-establish differentiation cocktail containing BMP4 in combination with activin/nodal and FGF2 signaling inhibitors in low O_2_ conditions was shown to allow TR differentiation, ensuring high efficiency, and supporting and extending to the porcine species data previously reported for the human.

The TR *in vitro* model generated in the present experiments may find useful applications in order to increase our knowledge on embryo implantation mechanisms and to better elucidate developmental disorders based on TR defects. It may also represent a valuable tool for drug discovery and veterinary regenerative medicine.

## Data Availability Statement

The original contributions presented in the study are included in the article/supplementary material, further inquiries can be directed to the corresponding author.

## Author Contributions

SA performed the experiments. GP performed the experiments and drafted the manuscript. FG and TB designed and coordinated the study and drafted the manuscript. All authors contributed to the article and approved the submitted version.

## Conflict of Interest

The authors declare that the research was conducted in the absence of any commercial or financial relationships that could be construed as a potential conflict of interest.
